# Implementing an established musculoskeletal educational curriculum in a new context: a study of effectiveness and feasibility

**DOI:** 10.1080/10872981.2020.1760466

**Published:** 2020-05-07

**Authors:** Meg Pearson, Andrea M. Barker, Michael J. Battistone, Stephen Bent, Krista Odden, Bridget O’Brien

**Affiliations:** a Department of Medicine, University of California, San Francisco, CA, USA; b San Francisco Veterans Affairs Health Care System, San Francisco, CA, USA; c Center of Excellence in Musculoskeletal Care and Education, Salt Lake City Veterans Affairs Medical Center, Salt Lake City, CT, USA; d Department of Family and Preventive Medicine, University of Utah, Salt Lake City, UT, USA; e Department of Internal Medicine, Division of Rheumatology, University of Utah, Salt Lake City, UT, USA; f Psychiatry, Epidemiology & Biostatistics, University of California, San Francisco, CA, USA; g School of Nursing, University of California, San Francisco, CA, USA; h Department of Medicine, Education Scientist, Center for Faculty Educators, University of California, San Francisco, CA, USA

**Keywords:** Musculoskeletal, primary care education, shoulder and knee exam, curriculum evaluation, graduate medical education

## Abstract

**Background**: Musculoskeletal (MSK) problems are common, yet many primary care (PC) providers feel inadequately trained to manage these conditions. Previous studies describe successful MSK educational innovations at single sites, but none have reported on subsequent attempts to replicate or adapt these innovations to new contexts. This article presents a study of a national Veterans Affairs MSK training program modified to fit an existing PC educational program.

**Objectives**: (1) To evaluate the effectiveness and feasibility of an adapted MSK curriculum in a new context. (2) To provide a model for adaptation studies in health professions education.

**Design**: A national MSK shoulder and knee curriculum was adapted for San Francisco VA PC trainees, which included a small-group workshop and workplace learning within a newly-created MSK clinic. Effectiveness was evaluated by assessments of trainee confidence in exam and injection skills (via 5-point Likert scale) and faculty-observed performance of knee and shoulder exams (reported as percent of maximum possible score). Feasibility was evaluated by determining acceptability of the program to PC trainees (via 5-point Likert scale) and ability to implement the curriculum using local resources.

**Results**: 52 trainees completed the training during a 2-year period. Trainees’ confidence in MSK exam skills improved from 3.3 to 4.5 for shoulder, and from 3.5 to 4.6 for knee. Confidence performing joint injections improved from 2.6 to 4.2 (shoulder) and 2.5 to 4.5 (knee) (p < 0.001 for all). Observed performance improved markedly – from 50% to 92% for shoulder, and 57% to 90% for knee. Feasibility was evident in high acceptability (5.0 for MSK clinic, and 4.9 for workshops), and successful and sustained implementation.

**Conclusions**: Adapting an established MSK curriculum to a new context was effective and feasible. This may serve as a more efficient model for improving trainee education than de novo curriculum design at individual sites.

## Introduction

Musculoskeletal (MSK) disorders affect more than 50% of the US population age 18 and over, nearly 75% age 65 and over, and are one of the leading reasons for visits to health-care providers [1,2]. Although MSK disorders such as chronic knee and shoulder pain are common in primary care settings, primary care providers (PCPs) often do not feel comfortable performing MSK examinations or therapeutic joint injections [3–6]. The 2011 US Bone and Joint Initiative Summit called upon health professions training programs to emphasize competency in the diagnosis and management of MSK conditions[7].

In 2012, a multi-method curriculum for evaluation and management of MSK problems commonly encountered in primary care settings was developed for PCPs and trainees at the Salt Lake City Veterans Affairs (SLCVA) Medical Center and University of Utah [8–10]. The following year, faculty from the San Francisco VA (SFVA) worked in partnership with SLC faculty to present this program for PCPs at SFVA[10]. The success of this program formalized a collaborative educational partnership between the two sites and galvanized the SFVA team to develop a similar MSK curriculum tailored to the needs of the local primary care training program.

The need for improved MSK training across the medical education continuum is well-established, and many educational models at the undergraduate and graduate primary care levels have been reported [5,8,11–13]. To our knowledge, this project is the first to describe the experience of adapting an established MSK curriculum to fit the resources and needs of another site. In this paper we aim to evaluate the effectiveness and feasibility of an adapted MSK curriculum in a new context and to provide a model for adaptation studies in health professions education.

## Materials and methods

### Original curriculum

We used an established MSK curriculum – a national Veterans Affairs MSK training program based at the SLCVA – as the starting point for our adapted curriculum. The SLCVA MSK curriculum has two versions: a three-day traveling Continuing Professional Development (CPD) curricula for PCPs that the authors completed when it came to the SFVA in 2013, as well as an on-site monthly ‘MSK Education Week’ for SLCVA trainees. Table 1 displays the core components of the two SLCVA MSK curricula. Curricular details and evaluation tools have been reported [8–10].

**Table 1.** Comparison of Original SLCVA Curricula vs Adapted SFVA Curriculum

Core Curricular Components	National SLCVA CPD curriculum	SLCVA ‘MSK Education Week’ curriculum	Adapted SFVA Primary Care trainee curriculum
Purpose	Improve MSK skills of practicing VA PCPs	Improve MSK skills of multidisciplinary trainees	Improve MSK skills of primary care trainees
Participants (Target learners)	Practicing PCPs	SLCVA trainees (IM, PM&R & orthopedic residents, PA and NP trainees)	SFVA primary care trainees (IM residents and NP trainees)
Faculty	Primary Care Internist, Rheumatologist, Orthopedic PA, Orthopedist, Endocrinologist	Primary Care Internist, Rheumatologist, Orthopedic PA, Orthopedist, Endocrinologist	Primary Care Internists and NP, PT. Workshop small group faculty also included rheumatologists
Structure & Duration of Curriculum	Consecutive 3-day curriculum SLCVA faculty travelled to partnering VA local sites	Consecutive 5-day curriculum delivered monthly at SLCVA	~ 2 day total curriculum delivered in increments over a year: 3–4 half-day teaching clinics held weekly + 1 four-hour workshop
Content	Emphasizes shoulder and knee management (and injection practice with models only); also includes back pain, bone health, general rheumatology	Emphasizes shoulder and knee management and injection skills (clinical practice, real patients); also includes back pain, bone health, general rheumatology	Shoulder and Knee Management and Injections (clinical practice, real patients)
Educational Activities	Lectures, group practice. *No workplace/clinic component*	Lectures, group practice, workplace learning in multiple clinics (Rheumatology, Orthopedic, PM&R, Primary Care MSK, Multidisciplinary MSK)	Lectures, group practice, workplace learning in a single clinic (Primary Care MSK)
Evaluation of Program	-Demographics (number of learners and credential) -Learner self-assessment of confidence in exam and injection skills -Faculty-rating of knee and shoulder exam skills (post- (in CPD Program))	-Demographics (number of learners and program of study) -Trainee self-assessment of confidence in exam and injection skills - Faculty-rating of knee and shoulder exam skills (post- (in MSK Week)) - Trainee rating of MSK Week	-Demographics (number of learners and program of study) -Trainee self-assessment of confidence in exam and injection skills -Faculty-rating of knee and shoulder exam skills (pre- and post- (in clinic)) -Trainee rating of MSK clinic and workshops

Key: CPD = Continuing Professional Development; IM = Internal Medicine; PA = Physician Assistant

PM&R = Physical Medicine and Rehabilitation; PT = Physical therapy; NP = Nurse Practitioner

### Description of adaptation process

Adapting the original MSK curricula to our local program included the following steps: 1) identifying essential elements of the original curriculum, 2) designing an adapted version of these elements, customized to fit the needs of our learners and optimizing use of our local resources (educator time and skills, space, materials) and 3) creating a plan to evaluate effectiveness and feasibility.

#### Step 1: Identifying essential elements of the original curriculum

We reviewed both SLCVA MSK curricula in detail. Based on guiding principles for effective MSK teaching [14,15] we identified 3 key components of the SLCVA curricula: 1) workplace learning in **MSK clinic**, which afforded the opportunity to gain and apply skills in the care of patients; 2) workshops emphasizing **small-group practice** (exam practice sessions and simulated case-based learning); and 3) high-yield **memory aids** (such as videos, checklists and handouts), which highlighted key teaching points during the sessions and clinics themselves, and allowed for easy reference when applying the skills to one’s clinical practice.

#### Step 2: Designing an adapted version of the curriculum to match the local context

The adapted SFVA primary care MSK curriculum teaches shoulder and knee exam, disease management and joint injections skills to SFVA primary care trainees, which include 2^nd^ and 3^rd^ year IM residents, NP students and NP residents. Table 1 highlights the curricular changes we made to align with the resources available within our local program: we narrowed the content to focus only on shoulder and knee conditions (and not back pain, osteoporosis and general rheumatology topics such as RA), and we limited our target learners to SFVA primary care trainees. This allowed us to pare down the curriculum to 2 days total (non-continuous), as described in more detail below. The decision to prioritize shoulder and knee conditions was based on several factors including 1) shoulder and knee steroid injections are commonly performed by PCPs and are important skills for our primary care trainees to acquire (contrasted to epidural steroid injections performed for back pain, for example, which are typically performed under imaging by subspecialists); 2) shoulder and knee pain are prevalent in our veteran population; and 3) there was a clinical need at our institution to improve patient access to care for shoulder and knee pain (whereas there was already a dedicated Spine Clinic and Pain Management Clinic at the SFVA to address back pain).

### Workplace learning: MSK clinic

We created a new MSK Clinic at the SFVA, which is housed within our existing primary care clinic and is staffed by two internists, one primary care nurse practitioner (NP), and one physical therapist. Trainees include 2^nd^ and 3^rd^ year Internal Medicine (IM) residents, NP students and NP residents. The SLCVA features a remarkable multidisciplinary MSK clinic, in which faculty and trainees from primary care, rheumatology, orthopedics, physical medicine and rehabilitation, and endocrinology all see patients together in a shared clinic[8]. Though ideal for patient care and education, we recognized that the integration of multiple distinct disciplines into a shared MSK clinic – with the attendant challenges of space limitations, scheduling, and faculty release time – was not feasible for the first iteration of our MSK clinic.

Patients are referred to the MSK clinic for evaluation and management of shoulder and knee pain, often including consideration for joint injections. The first year of the MSK clinic did not include trainee education but rather focused on streamlining clinic operations processes – including patient referral and scheduling processes, in collaboration with rheumatology, orthopedics and primary care clinic leadership. Trainees were added in year 2.

Trainees attend MSK clinic one half-day per week during month-long outpatient blocks, with a goal of completing 3–4 half-day sessions and performing at least five subacromial and five knee injections. Trainees see patients one-on-one with MSK faculty in the exam room, as opposed to the usual precepting model in which multiple trainees present cases to a few faculty preceptors in a separate conference room. The MSK clinic provided trainees with repeated exposure to patients with shoulder and knee problems, and the one-on-one precepting model allowed for real-time coaching from expert faculty on the relevant history, physical exam, clinical management, patient education, and joint injection skills.

### Workshops with emphasis on small-group practice sessions

Trainees participate in a four-hour interactive workshop focusing on anatomy, exam skills and management of common shoulder and knee problems. Each workshop includes four sections for both shoulder and knee: 1) lecture on anatomy and exam, 2) small-group exam practice sessions facilitated by expert faculty, 3) lecture on diagnosis and management of common shoulder/knee conditions, and 4) small-group faculty-led case simulations.

Workshops are presented twice a year so that IM residents can participate during an outpatient block (residents rotate one month on/off outpatient block). Each workshop includes 5–6 faculty members (the 3 MSK Clinic providers and physical therapist and 1–3 rheumatologists and/or sports medicine experts) and 20–30 trainees.

### Memory aids

Trainees receive shoulder and knee physical exam checklists and corresponding videos via email attachments prior to their first MSK clinic session. These materials, in addition to succinct clinical management handouts, are also reinforced in the workshops.

#### Step 3: designing an evaluation plan

We designed a plan to evaluate the *effectiveness* of the adapted MSK curriculum in improving trainees’ MSK skills and its *feasibility* within our primary care training program. We reviewed the program evaluation tools used in the SLCVA programs and the published evidence of their validity, then modified these for congruence with our adapted curriculum [8–10,16]. To evaluate the *effectiveness* of the adapted MSK curriculum we assessed 1) trainees’ confidence in exam and injection skills and 2) their observed performance of knee and shoulder exams pre- and post-curriculum. For the latter, we performed standardized faculty observations of shoulder and knee exams during real patient encounters in the MSK clinic (on trainees’ first and last clinic sessions), using an adaptation of the SLCVA checklists.

To evaluate the *feasibility* of the adapted MSK curriculum, we assessed 1) the acceptability of the MSK curriculum to primary care trainees as expressed by ratings (0 to 5-point Likert scale) and free-text feedback comments and 2) the ability to implement the adapted curriculum using resources available within our primary care training program and home institution.

This project was reviewed by the Institutional Review Board of the University of California, San Francisco and the VA Office of Research and Development and was classified as exempt.

## Results

### Effectiveness

From September 2014 to December 2016, 52 trainees rotated through the MSK clinic (41 IM residents and 11 NP trainees). Paired pre- and post-course surveys and exam scores were included for analysis, resulting in 26 paired self-assessments and 32 paired observed exams (response rates of 50% and 62%, respectively). We did not include any unmatched pre- and post-curriculum surveys or exam scores, which were unmatched due to either missing confidential trainee IDs or missing one or both surveys.

Mean self-assessment ratings of confidence are shown in Table 2. All measures of confidence improved significantly following the curriculum. Trainees’ confidence in MSK exam skills improved from 3.3 to 4.5 for shoulder, and from 3.5 to 4.6 on knee (on a 5-point Likert scale; p < 0.001). Confidence performing joint injections improved from 2.6 to 4.2 (shoulder) and 2.5 to 4.5 (knee).

**Table 2.** Mean self-assessment ratings pre- and post-course participation^a.^

Question	Pre-Course Mean (SD)	Post-Course Mean (SD)	Mean Difference
I can perform a *physical examination* of the *shoulder* that allows me to diagnose common causes for shoulder pain	3.3 (0.8)	4.5 (0.5)	1.2^b^
I can perform a successful *subacromial space injection*	1.8 (0.9)	4.2 (1.1)	2.4^b^
I can perform a *physical examination* of the *knee* that allows me to diagnose common causes for knee pain	3.5 (0.7)	4.6 (0.5)	1.1^b^
I can perform a successful *intra-articular knee injection*	2.5 (1.1)	4.5 (0.9)	2.0^b^

^a^Mean numerical scores on a 5-point scale (1 = strongly disagree; 2 = disagree; 3 = neutral; 4 = agree; 5 = strongly agree); N = 26

^b^p < 0.001 compared with pre-course results.

Faculty observed performance of the shoulder and knee exam increased markedly (Table 3). Mean scores (% exam checklist items performed correctly) improved from 50% to 92% for shoulder, and from 57% to 90% for knee (p < 0.001). The post-curriculum scores for the SFVA adapted MSK curriculum were similar to those seen in the SLCVA curriculum for PCPs – 90% for shoulder and 86% for knee[9].

**Table 3.** Observed performance of the physical examination pre- and post-course participation^a^

Physical Exam	Pre-Course Mean % (SD)	Post-Course Mean % (SD)	Mean Difference
Shoulder (n = 33)	50.1 (15.7)	91.9 (7.1)	41.8^b^
Knee (n = 32)	57.0 (14.6)	89.5 (10.3)	32.5^b^

^a^Scores calculated as percent of maximum score.

^b^p < 0.001 compared with pre-course results.

### Feasibility

#### Acceptability

The adapted SFVA MSK curriculum showed high acceptability among primary care trainees, as reported in Table 4. Overall mean ratings for clinics and workshops were 5 and 4.9 (sd 0.3) out of 5, respectively. In written feedback, trainees frequently cited the hands-on practice with experts, in both clinic and workshops, as highlights of the curriculum. Trainees’ comments included: ‘I felt like I walked away with real tangible skills that I’ve already used in my clinic,’ ‘This was my favorite clinic in residency!’ and ‘Probably the best thought out, highest yield clinic I’ve participated in this year. Just fantastic!’

**Table 4.** Acceptability ratings of clinical and interactive workshop experiences^a.^

	Min	Max	Mean (SD)
**Clinical Experience^b^**			
Overall quality of teaching by MSK faculty	5	5	5.0 (0.0)
Quality of feedback on specific MSK skills	4	5	5.0 (0.2)
Overall quality of the MSK clinical experience	5	5	5.0 (0.0)
Please estimate the likelihood that you will make changes in your clinical activities as a result of the MSK clinical experience	4	5	5.0 (0.2)
**Workshop Experience^c^**			
Overall rating of workshop	4	5	4.9 (0.3)

^a^Mean numerical scores on a 5-point scale (1 = poor; 2 = fair; 3 = good; 4 = very good; 5 = excellent)

^b^N = 35

^c^N = 23; Results for overall workshop ratings only available for 2016

#### Ability to implement the adapted curriculum using local resources

We were able to implement the curriculum as planned using resources available within our home institution. During the first year, we successfully pursued a one-year Interprofessional Education Instructional Grant[17], effective July 2014, to support the faculty effort required. The MSK curriculum has since been sustained without the need for external funding. We gained institutional and departmental support for the MSK clinic by positioning our clinic to meet an identified need to provide timely specialty care for MSK complaints at our facility (by off-loading both orthopedics and rheumatology clinics), as well as a need for improved MSK training within primary care education[18]. Gaining buy-in from institutional leadership enabled us to secure the clinical space and faculty release time, as well as trainee time, needed for the new MSK clinic. Because MSK clinic is housed within our primary care clinic, we needed no additional administrative support; staff were able to incorporate scheduling and check-in for the MSK clinic into their workflow. Colleagues in our institution’s Office of Medical Education assisted with study design, evaluation, and data analysis.

## Discussion

Consistent with prior studies, our project confirms that our primary care trainees have low baseline confidence and ability in MSK exam, management, and joint injections [6,8]. Our study suggests that our adapted primary care MSK curriculum was effective in improving trainee confidence and clinical skills, and that the process of implementing existing curricula in a new context was feasible and sustainable. Our experience adapting the more comprehensive and time-intensive SLCVA MSK curricula to our targeted version has led us to believe that the three key elements of an effective MSK educational curriculum within a primary care training program are 1) creating an **MSK clinic**, which creates opportunities for trainees to care for patients with MSK complaints under expert supervision, 2) workshops with **small-group practice sessions** and 3) **memory aids** that provide take-home summaries that allow for easy reference in subsequent clinical practice. Educators seeking to implement a new MSK curriculum at their site may find it helpful to review the original SLCVA MSK curricula and our adapted version to create a best-fit for their own context.

To assess the feasibility of adapting an MSK educational curriculum to new sites, it is helpful to evaluate the acceptability of the curriculum to the target learners, and the ability to implement the adapted curriculum using available resources. The resource utilization strategies that we used to make the project feasible are likely available to other primary care training programs seeking to implement a similar Primary Care MSK clinic and accompanying curriculum, including *gaining institutional support* by highlighting the role of a Primary Care MSK clinic in improving access to specialty care for MSK conditions; *leveraging existing clinical administrative support* by housing MSK clinic within the physical space and staffing pool of the primary care clinic if possible; and *drawing upon the educational expertise of colleagues* within the primary care training program or affiliated institution for assistance with study design, implementation, and evaluation.

In addition to gaining an understanding of the essential components of an effective primary care MSK educational curriculum and the feasibility of adapting these curricula to new contexts, our paper offers a general model for adaptation of educational interventions. Innovations abound in health professions education, as evidenced by journals and journal sections dedicated to dissemination of such work. Many of these describe experiences at a single site [19,20] with the hope that others will recognize a gap and implement something similar in their own context. While this type of replication with adaptation likely occurs, such efforts are rarely published [21–23]. Consequently, educators lack valuable information that could guide efforts to adapt and implement innovations in new contexts. The model for adaptations of educational interventions includes a) identifying key components of the original interventions, b) designing an adapted version of the intervention to match the local context and c) evaluating the intervention’s efficacy and feasibility in the new context. The evaluation component may be particularly important for examining the strengths and weaknesses of an intervention and providing evidence to gain future financial support and resources for continuation.

Our study has several limitations. Only half the trainees who participated in the intervention completed both pre- and post-assessments, which could bias our findings if those who completed pre-post assessments differ from those who did not. We have no reason to suspect such differences. The low response rates were due to a number of factors, including missing confidential participant IDs on some forms, lack of a tracking system for trainees whose participation often spanned several months, and the general hectic pace of the life of a medicine resident. We believe response rates could be improved in future studies by having a research staff member dedicated to tracking and promoting timely form completion. Without a comparison group of non-participants, we cannot rule out the possibility of improvement without intervention. Informal reports from residents suggest that their MSK skills do not improve markedly during standard residency training. We did not administer follow-up assessments to determine sustainability of the MSK skills, which is a goal for future research for our team. Lastly, we have not collected validity evidence on our assessment instruments, but we selected them based on alignment with the goals of our intervention.

Adapting a successful educational intervention can make the process of curriculum or program development much more efficient for busy clinician educators who typically have little protected time for creative educational initiatives. In addition to the obvious benefit of shared curricular materials, our educational partnership with the SLCVA was an invaluable resource for our nascent primary care MSK program. We sought guidance from the SLCVA team on a range of issues, from clinical MSK topics to clinic operations to program evaluation. Sharing successful MSK interventions across primary care training programs may provide an efficient means for health professions training programs to achieve trainee competency in the diagnosis and management of MSK conditions.

## Supplementary Material

Supplemental Material

## Prior abstract or poster presentation

This work was presented as poster presentations at the 2015 UCSF Education Showcase, the 2016 California Regional Society of General Internal Medicine Annual Meeting and the 2016 California Macy Regional Conference on Innovations in Graduate Medical Education.

## References

[1] The Burden of Musculoskeletal Disease in the USA; 2011. Available at: http://www.boneandjointburden.org/

[2] Schappert SM, Rechtsteiner EA. Ambulatory medical care utilization estimates for 2007. Vital Health Stat. 2011;13(169):1–7. 21614897

[3] Jolly M, Curran JJ. Underuse of intra-articular and periarticular corticosteroid injections by primary care physicians: discomfort with the technique. J Clin Rheumatol. 2003;9(3):187–192. 17041456 10.1097/01.RHU.0000073587.90836.23

[4] Houston TK, Connors RL, Cutler N, et al. A primary care musculoskeletal clinic for residents: success and sustainability. J Gen Intern Med. 2004;19(5 Pt 2):524–529. 15109317 10.1111/j.1525-1497.2004.30173.x PMC1492313

[5] Vogelgesang SA, Karplus TM, Kreiter CD. An instructional program to facilitate teaching joint/soft-tissue injection and aspiration. J Gen Intern Med. 2002;17(6):441–445. 12133158 10.1046/j.1525-1497.2002.10310.x PMC1495064

[6] Freedman KB, Bernstein J. Educational deficiencies in musculoskeletal medicine. J Bone Joint Surg Am. 2002;84-A(4):604–608. 10.2106/00004623-200204000-00015 11940622

[7] Gnatz SM, Pisetsky DS, Andersson GB. The value in musculoskeletal care: summary and recommendations. Pm R. 2012;4(5):379–382. 22613364 10.1016/j.pmrj.2012.04.004

[8] Battistone MJ, Barker AM, Grotzke MP, et al. Effectiveness of an interprofessional and multidisciplinary musculoskeletal training program. J Grad Med Educ. 2016;8(3):398–404. 27413444 10.4300/JGME-D-15-00391.1 PMC4936859

[9] Battistone MJ, Barker AM, Grotzke MP, et al. “Mini-residency” in musculoskeletal care: a national continuing professional development program for primary care providers. J Gen Intern Med. 2016;31(11):1301–1307. 27350280 10.1007/s11606-016-3773-4 PMC5071283

[10] Battistone MJ, Barker AM, Lawrence P, et al. Mini-residency in musculoskeletal care: an interprofessional, mixed methods educational initiative for primary care providers. Arthritis Care Res. 2016;68(2):275–279. 10.1002/acr.22644 26097001

[11] Hose MK, Fontanesi J, Woytowitz M, et al. Competency based clinical shoulder examination training improves physical exam, confidence, and knowledge in common shoulder conditions. J Gen Intern Med. 2017;32(11):1261–1265. 28785987 10.1007/s11606-017-4143-6 PMC5653557

[12] Wilcox T, Oyler J, Harada C, et al. Musculoskeletal exam and joint injection training for internal medicine residents. J Gen Intern Med. 2006;21(5):521–523. 16704403 10.1111/j.1525-1497.2006.00442.x PMC1484790

[13] Denizard-Thompson N, Feiereisel KB, Pedley CF, et al. Musculoskeletal basics: the shoulder and the knee workshop for primary care residents. MedEdPortal. 2018;14(1):10749. DOI:10.15766/mep_2374-8265.10749. 30800949 PMC6342436

[14] Lawry GV 2nd, Schuldt SS, Kreiter CD, et al. Teaching a screening musculoskeletal examination: a randomized, controlled trial of different instructional methods. Acad Med. 1999;74(2):199–201. 10065062 10.1097/00001888-199902000-00020

[15] O’Dunn-Orto A, Hartling L, Campbell S, et al. Teaching musculoskeletal clinical skills to medical trainees and physicians: a best evidence in medical education systematic review of strategies and their effectiveness: BEME Guide No. 18. Med Teach. 2012;34(2):93–102. 22288986 10.3109/0142159X.2011.613961

[16] Battistone MJ, Barker AM, Beck JP, et al. Validity evidence for two objective structured clinical examination stations to evaluate core skills of the shoulder and knee assessment. BMC Med Educ. 2017;17(1):13. 28086879 10.1186/s12909-016-0850-7 PMC5237332

[17] Adler SR, Chang A, Loeser H, et al. The impact of intramural grants on educators’ careers and on medical education innovation. Acad Med. 2015;90(6):827–831. 25760956 10.1097/ACM.0000000000000685

[18] Gnatz SM, Pisetsky DS, Andersson GB. The value in musculoskeletal care: summary and recommendations. Pm R. 2012;4(5):379–382. 22613364 10.1016/j.pmrj.2012.04.004

[19] Reed DA, Beckman TJ, Wright SM, et al. Predictive validity evidence for medical education research study quality instrument scores: quality of submissions to JGIM’s medical education special issue. J Gen Intern Med. 2008;23(7):903–907. 18612715 10.1007/s11606-008-0664-3 PMC2517948

[20] Sullivan GM. Deconstructing quality in education research. J Grad Med Educ. 2011;3(2):121–124. 22655130 10.4300/JGME-D-11-00083.1 PMC3184904

[21] Varpio L, Bell R, Hollingworth G, et al. Is transferring an educational innovation actually a process of transformation? Adv Health Sce Educ. 2012;17(1):357–367. 10.1007/s10459-011-9313-4 21725841

[22] Fabry G, Fischer MR. Replication–The ugly duckling of science? GMS Z Med Ausbild. 2015;32(5):57. 10.3205/zma000999 PMC4647164 26604999

[23] Artino AR Jr. Why don’t we conduct replication studies in medical education? Med Educ. 2013;47(7):746–747. 23683109 10.1111/medu.12204

