# Supplementary material for: Implementing an established musculoskeletal educational curriculum in a new context: a study of effectiveness and feasibility
Source: Med Educ Online. 2020 May 7;25(1):1760466. doi: 10.1080/10872981.2020.1760466 (PMC7241557; doi:10.1080/10872981.2020.1760466)
Supplement: Supplemental Material [file ZMEO_A_1760466_SM0260.zip › Supplemenatry/Supplemental--Knee Exam Checklist.docx]

Name Preceptor/Date

Knee Physical Examination __Pre-test __Post-test

Trainee: NP Student  NP Resident  MD Resident  Other (please specify):______________

|  | **Examination** | **Performed** | | | **Technique Adequate** |
| --- | --- | --- | --- | --- | --- |
| **1** | ***Observation*** |  | | |  |
|  | Standing | 0 | 1 | 2 | Gait, alignment, popliteal fossa, |
|  | Supine position, knee adequately exposed | 0 | 1 | 2 | Alignment, atrophy, lesions, scars, erythema |
| **2** | ***Palpation – knee extended*** |  |  |  |  |
|  | Effusion | 0 | 1 | 2 | Full extension, medial/lateral gutters |
|  | Quadriceps tendon | 0 | 1 | 2 |  |
|  | Patellar tendon | 0 | 1 | 2 |  |
|  | Tibial tubercle | 0 | 1 | 2 |  |
|  | Patellar facets | 0 | 1 | 2 |  |
|  | Patellar compression (grind) test | 0 | 1 | 2 |  |
| **3** | ***Range of Motion*** |  | | |  |
|  | Extension/Flexion 0-140° | 0 | 1 | 2 |  |
|  | Hip IR (30°) and ER (60°) |  |  |  |  |
| **4** | ***Palpation- knee flexed 90°*** |  | | |  |
|  | Flex to 90° with heel resting on table | 0 | 1 | 2 |  |
|  | Medial joint line | 0 | 1 | 2 |  |
|  | Medial Collateral Ligament (MCL) | 0 | 1 | 2 | MFE/Medial Tibia |
|  | Pes anserine bursa | 0 | 1 | 2 |  |
|  | Lateral joint line | 0 | 1 | 2 |  |
|  | Lateral Collateral Ligament (LCL) | 0 | 1 | 2 | LFE/ Fibular Head |
|  | Iliotibial band (Noble Compression Test) |  |  |  | Palpate lateral femoral epicondyle Passive knee ROM (pain at 30°) |
| **5** | ***Stability/Provocative Testing*** |  | | |  |
|  | Anterior Cruciate Ligament Anterior Drawer | 0 | 1 | 2 | Knee flexed to 90 degrees Examiner stabilizes foot  Thumbs on anterior tibia, translate anterior |
|  | Posterior Cruciate Ligament  Posterior Drawer | 0 | 1 | 2 | Knee flexed to 90 degrees Examiner stabilizes foot  Thumbs on anterior tibia, translate posterior |
|  | Anterior Cruciate Ligament Lachman’s Test | 0 | 1 | 2 | Knee flexed to 30 degrees Hands near joint line Anterior tibial translation |
|  | Medial Collateral Ligament (Valgus stress) | 0 | 1 | 2 | 30 degrees flexion |
|  | Lateral Collateral Ligament (Varus stress) | 0 | 1 | 2 | 30 degrees flexion |
|  | Medial Meniscus (McMurray Test) | 0 | 1 | 2 | Fingers on posteromedial joint line Full knee flexion  External rotation sweep and slow leg extension |
|  | Lateral Meniscus (McMurray Test) | 0 | 1 | 2 | Fingers on posterolateral joint line Full knee flexion  Internal rotation sweep and slow leg extension |
|  | Thessaly |  |  |  | Stand flat footed on one leg  Flex knee to 20°, rotate femur on tibia medially & laterally |

Total score: ______/44_

SC


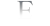

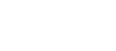

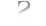

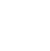

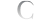

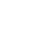

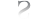

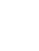

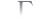

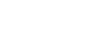


Key: 0 = Not performed 1 = Performed but incorrect 2 = Performed without error

E

| **6** | ***Neurologic Testing (if lumbar radiculopathy suspected)*** |  |  |
| --- | --- | --- | --- |
|  | Motor |  |  |
|  | Knee extension (L3) |  |  |
|  | Knee flexion (S2) |  |  |
|  | Ankle dorsiflexion (L4) |  |  |
|  | Ankle plantarflexion (S1) |  |  |
|  | Great toe extension (L5) |  |  |
|  | Reflexes |  |  |
|  | Patella (L3, L4) |  |  |
|  | Achilles (S1, S2) |  |  |
|  | Sensation |  |  |
|  | Anteromedial thigh (L3) |  |  |
|  | Anterolateral thigh (L4) Great toe |  |  |
|  | Dorsum of foot (L5) |  |  |
|  | 5th toe (S1) |  |  |
|  | (S2)  Posteromedial lower leg |  |  |
