# Supplementary material for: Implementing an established musculoskeletal educational curriculum in a new context: a study of effectiveness and feasibility
Source: Med Educ Online. 2020 May 7;25(1):1760466. doi: 10.1080/10872981.2020.1760466 (PMC7241557; doi:10.1080/10872981.2020.1760466)
Supplement: Supplemental Material [file ZMEO_A_1760466_SM0260.zip › Supplemenatry/Supplemental--Shoulder Exam Checklist.docx]

Name Preceptor/Date

Shoulder Physical Examination __Pre-test __Post-test

Trainee**:**  NP Student  NP Resident  MD Resident  Other (please specify):______________

|  | **Examination** | | **Performed** | | | | | | | | **Technique Adequate** |
| --- | --- | --- | --- | --- | --- | --- | --- | --- | --- | --- | --- |
| **1** | ***Observation*** | |  | | | | | | | |  |
|  | Adequate exposure | | 0 | 1 | 2 | | | | | | Observe as they disrobe for degree of discomfort |
|  | Examine for symmetry, scars, skin lesions, erythema, edema | | 0 | 1 | 2 | | | | | |  |
|  | Examine for atrophy (supraspinatus, infraspinatus, deltoid) | | 0 | 1 | 2 | | | | | |  |
| **2** | ***Palpation*** | |  | | | | | | | |  |
|  | Acromioclavicular joint | | 0 | 1 | 2 | | | | | |  |
|  | Biceps tendon | | 0 | 1 | 2 | | | | | |  |
|  | Coracoid | | 0 | 1 | 2 | | | | | |  |
|  | Subacromial space | | 0 | 1 | 2 | | | | | | Lateral and posterolateral |
| **3** | ***Range of Motion*** *(Note: check passive ROM if active is limited.* | |  | | | | | | | |  |
| **4** | *This will identify mechanical block vs. shoulder weakness).*  ***Motor Function of Rotator Cuff*** | |  |  |  |  |  |  |  |  |  |
| Supraspinatus | ROM: Active abduction in scapular plane  Painful arc (60-120°) | | 0 | 1 | 2 | | | | | | Normal 0-180°  Scapular plane  Neutral rotation (thumbs to ceiling) |
|  | Motor:  Drop arm test  Empty Can Test | | 0 | 1 | 2 | | | | | | Scapular plane  Full pronation (thumbs to floor) Resisted abduction at 90° or less |
| Infraspinatus/  Teres Minor | ROM: Active external rotation | | 0 | 1 | 2 | | | | | | 0-90°/normal at least 30°  Elbows at side |
|  | Motor:  Active external rotation against resistance  Passive ER lag test | | 0 | 1 | 2 | | | | | | Elbows at side  Start with hands near midline  Elbow to 90°; elevated 20° scaption  Passively external rotate to end range |
| Subscapularis | ROM: Active internal rotation along spine  (Observe patient from behind) | | 0 | 1 | 2 | | | | | | T3 = Scapular spine  T7 = Inf angle of scapula  L4 = Iliac crest |
|  | Motor:  Lift Off Test  Passive IR lag test | | 0 | 1 | 2 | | | | | | Hand at lumbar spine  Actively lifts arm off back against resistance at wrist  Arm in maximum internal rotation  Dorsum of hand passively lifted away from lumbar spine |
| **5** | | ***Provocative Testing*** |  | | | | | | |  | |
|  | | ***Biceps Testing*** |  | | | | | | |  | |
|  | | Speed’s Test | 0 1 2 | | | |  |  | | 60° forward elevation  Hand in supination  20-30° elbow flexion  Apply downward pressure to forearm | |
|  | | Yergason’s Test | 0 1 2 | | | |  |  | | Elbow at side, 90° flexion Palm in supination  Resisted supination | |
|  | | ***Impingement Testing*** |  | | | | | | |  | |
|  | | Neer’s Test | 0 1 2 | | |  | | |  | Elbow extended  Full pronation  Maximal passive forward elevation of shoulder with scapular stabilization | |
|  | | Hawkin’s Test | 0 1 2 | | |  | | |  | Shoulder 90° abduction  Scapular plane  90° elbow flexion  Internal rotation + horizontal adduction | |
|  | | ***AC Joint Testing*** |  | | | | | | |  | |
|  | | Scarf Test (aka Cross-arm test) | 0 1 2 | | |  | |  | | Forward flex shoulder to 90°  Forced or Active horizontal adduction | |
|  | | ***Glenohumeral Stability*** |  | | | | | | |  | |
|  | | Apprehension  Relocation |  | | | | | | | Patient is supine  Abducted 90°  90° elbow flexion  Placed in maximal external rotation  Place posterior force on mid-humerus while externally rotating arm | |
|  | | Load and Shift |  | | | | | | | Patient is seated or supine  Arm slightly abducted 10°  Examiner stabilizes scapula  Grasp humeral head anterior & posterior  Axial load compresses humeral head into glenoid labrum  Shift anterior and posterior for laxity | |
|  | | ***Labral Testing*** |  | | | | | | |  | |
|  | | O’Brien |  | | | | | | | 90° forward elevation and maximal internal rotation  Horizontally adducted 10° thumb down  Apply downward pressure on the wrist  Repeat exam with resisted supination and thumb up | |
| **6** | | ***Neurologic Testing (if indicated)*** |  | | | | | | |  | |
|  | | Spurling’s Test |  | | | | | | | Slight neck extendsion toward affected side Apply axial compression. | |
|  | | Motor |  | | | | | | |  | |
|  | | Shoulder abduction, forearm supination (C5) |  | | | | | | |  | |
|  | | Elbow flexion, wrist extension (C6) |  | | | | | | |  | |
|  | | Elbow extension/wrist flexion/finger extension (C7) |  | | | | | | |  | |
|  | | Finger flexion/thumb abduction (C8) |  | | | | | | |  | |
|  | | Reflexes |  | | | | | | |  | |
|  | | Biceps (C5, C6) |  | | | | | | |  | |
|  | | Brachioradialis (C6) |  | | | | | | |  | |
|  | | Triceps (C7, C8) |  | | | | | | |  | |
|  | | Sensation |  | | | | | | |  | |
|  | | Deltoid (C5) |  | | | | | | |  | |
|  | | Radial aspect of arm/hand, thumb (C6) |  | | | | | | |  | |
|  | | 3rd digit (C7) |  | | | | | | |  | |
|  | | Ulnar aspect of arm/hand and 5th digit (C8) |  | | | | | | |  | |

Total score: _____/ 36

Key: 0 = Not performed 1 = Performed but incorrect 2 = Performed without error
